# Intrathecal injection of bone marrow concentrate in children with autism spectrum disorder: a retrospective chart analysis

**DOI:** 10.3389/fmed.2025.1666486

**Published:** 2025-09-18

**Authors:** Georg S. Kobinia, Adam Bukaty, Elisabeth Holly, Gloria Kobinia, Philipp R. Heuberer, Brenda Laky

**Affiliations:** ^1^Austrian Society of Regenerative Medicine, Vienna, Austria; ^2^Kobinia-Med, Institute for Regenerative Medicine, Vienna, Austria; ^3^Sigmund Freud University, Faculty of Medicine, Vienna, Austria; ^4^Austrian Research Group for Regenerative and Orthopedic Medicine (AURROM), Vienna, Austria

**Keywords:** autism spectrum disorders (ASD), autologous, bone marrow concentrate, intrathecal, stemcell therapy, children, autism treatment evaluation checklist (ATEC)

## Abstract

**Background:**

The growing prevalence of autism spectrum disorder (ASD) underscores the urgent need for therapies that target underlying biological mechanisms, with cell-based interventions offering a potentially transformative approach by targeting core physiological disruptions rather than providing temporary symptom management. The purpose of this study was to report on our experience with an autologous cell-based intervention in children with ASD.

**Methods:**

This retrospective data analysis included pre- and postinterventional data from 128 children with ASD who received intrathecal injections of autologous bone marrow concentrate. Patient and procedure related characteristics, complications, and the Autism Treatment Evaluation Checklist (ATEC) scores were extracted from patient's medical records.

**Results:**

Data were analyzed from 128 children (27 females and 101 males), aged between two and 16 years at their first intervention. A total of 32.8% underwent more than two single-step procedures. Significant improvements from the first to the second intervention were detected in the total and all subgroup ATEC scores, as well as in the severity groups (*p* < 0.001). Following the intervention, 4.6% of children transitioned from the “mild” or “moderate” to the “no symptoms” category, and 25.4% of the initially categorized “severe” group shifted to a milder symptom category. The average total ATEC score improved from the first to the second intervention by 19.0 ± 17.1 points, and one 60-point improvement was detected. The recorded ATEC score improvements in 85.9% of patients were similar between genders, as well as between age groups. A subgroup analysis of 39 patients who received three interventions also showed statistically significant differences in all ATEC scores between the three time points (*p* < 0.001). The highest improvements occurred after the first intervention, continued to improve over time, and remained reduced even three to four years after the intervention. There was not a single serious adverse event in the 307 treatments. All complications (e.g., nausea/vomiting) were resolved within a week or less after the procedure.

**Conclusion:**

Both a significant improvement in ATEC scores, and significant severity shifts to milder forms–even into the “no symptoms” category–suggest a measurable improvement in autism-related symptoms after autologous, bone marrow derived, intrathecally applied single procedures in children with ASD.

## 1 Introduction

Autism spectrum disorders (ASD) were first formally described by Leo Kanner in 1943 (1), marking a foundational moment in the clinical recognition of the condition. Although earlier accounts—such as those by Grunya Sukhareva in the 1920s—documented similar behavioral features, these insights had minimal impact on the development of mainstream clinical concepts at the time (2). Despite the decades that have passed since these initial descriptions, advances in elucidating the underlying causes and effective treatments of ASD remain modest, highlighting the persistent complexity of its etiology and pathophysiology. Hence, the disease is still considered incurable, as there is no established cure or treatment for its underlying pathophysiology.

While this was somewhat acceptable as long as the disease remained a rarity, the incidence of ASD has skyrocketed during the last 20 or 30 years. According to the latest data from the United States (U.S.) Centers for Disease Control and Prevention (CDC), approximately 1 in 31 children of age 8 has been identified with ASD (3), reflecting a continued rise in prevalence over recent years. In response to this growing public health concern, federal research initiatives, such as those supported by the National Institute of Mental Health (NIMH), have prioritized autism as a key focus area, aiming to better understand its etiology and pathophysiology, and to explore novel treatment strategies.

ASD is generally described as a neurodevelopmental condition with an onset early in life, and is characterized by impairments in communication, social interaction, cognitive awareness and behavior. While traditionally approached from a psychological perspective, there has been a significant paradigm shift toward understanding ASD as a biologically rooted brain disorder, with neuroinflammation emerging as a central pathophysiological mechanism (4). Indeed, environmental factors (e.g., heavy metals) are increasingly recognized as potential contributors to neuroinflammation in ASD, in part by impairing detoxification mechanisms and inducing epigenetic alterations during both prenatal development and later life stages (5–7). However, precision medicine, which customizes therapeutic strategies based on an individual's unique genetic, molecular, and cellular characteristics, has gained increasing relevance in ASD research and therapy (8). Thus, cell-based approaches are of growing interest, due to their immunomodulatory properties and ability to migrate to sites of inflammation (9).

Despite a steadily increasing prevalence of ASD in children (10) and the knowledge regarding the multifactorial etiology involving interactions between genetic, epigenetic, inflammatory, immunological, and environmental influences (11–16), clinical management remains a challenging task due to the heterogeneity of the disease. The diagnosis of ASD still relies on behavioral assessments, such as the Autism Diagnostic Observation Schedule (ADOS) and the Autism Diagnostic Interview-Revised (ADI-R), despite their subjective components (as they are based on observation and interviews performed by professionals). Although multiple biological pathways have been proposed as potential factors in ASD development, and despite extensive research efforts, no specific and universally validated biomarker has been identified for ASD either. Such biomarkers could serve not only as a diagnostic tool, but they may also allow for monitoring of therapeutic progress. Given the absence of validated biomarkers for ASD, behavioral rating scales remain essential for treatment monitoring. One such tool, the Autism Treatment Evaluation Checklist (ATEC), is particularly valuable because it offers a structured and quantifiable method to assess changes in core symptoms over time. It captures critical, caregiver-reported observations of day-to-day functional shifts, and it is sensitive enough to detect treatment effects–making it a reliable tool for both clinical practice and research settings. Its non-invasive, cost-effective and user-friendly design also makes it highly suitable for long-term tracking, especially in home or community-based environments where traditional clinical assessments may not be feasible. While diagnostic tests (e.g., ADOS, ADI-R) are performed by psychologists, psychiatrists, and/or neurologists, the ATEC score is completed by those who take care of the child on a 24/7 basis. Ultimately, those who provide continuous care for their child are best positioned to observe meaningful changes and assess treatment efficacy based on day-to-day functioning. While concerns have been raised about potential bias in caregiver-reported outcomes, particularly in self-financed interventions, our experience suggests the opposite: parents who invest in a therapeutic approach often demonstrate heightened critical scrutiny and are unlikely to accept superficial or illusory improvements.

However, therapeutic approaches have extended beyond behavioral and pharmacological interventions, which are predominantly symptomatic in nature, aiming to mitigate manifestations of ASD rather than addressing its underlying etiological and biological factors. Other strategies, such as dietary and nutritional supplementation, neuromodulation, neurostimulation, neurofeedback, and cell-based therapies aim to address underlying pathophysiological mechanisms, such as neuroinflammation, with the potential to restore or modulate normal biological functions rather than merely alleviating symptoms. Indeed, biological, cell-based approaches for ASD, which target inflammation and immune dysregulations, are on the rise, and various stem cell interventions have been previously applied in children with ASD (17–24).

There are different types of stem cell interventions for children with ASD, which differ in regards to cell source, including autologous (18, 19, 22–25) and allogeneic (17, 20, 24) procedures; cell types including, e.g., bone marrow (22, 23, 25), umbilical cord blood, and umbilical Wharton jelly mesenchymal cells (17–20, 24); cell processing variants, including culture expanded (17, 22), concentrated (gradient density centrifugation) (23, 25), unfractionated (18–20, 24), point-of-care (23) and single vs. multiple step procedures (17, 18, 20, 22–24); application methods such as single (18–20, 24, 25) or multiple (17, 20, 22, 23) applications; and various delivery routes such as intravenous (18–20, 24), intrathecal (22, 23, 25), or a combination of both intrathecal and intravenous (17).

The general aim of this retrospective analysis was to contribute to previous studies by reporting our experience with autologous bone marrow aspiration and intrathecal injection of bone marrow concentrate in a large number of children with ASD. The primary aim of this study was to identify and investigate possible ATEC score changes that may occur subsequent to this therapy and to track efficacy of treatments over time.

## 2 Methods

### 2.1 Study design and patient selection

Medical records of children with ASD who underwent their first autologous, bone marrow derived, intrathecally applied stem cell treatment—performed as a single step procedure by the first (GSK) and two other authors (PH, AB) from April 4th, 2019, until September 10th, 2024—were evaluated. Included were data sets of children with ASD between the ages of two and 18 years, regardless of gender, who received their first and second interventions at our institution. The medical records of children with ASD who were younger than 2 years of age and older than 18 years of age were not included. Patients who have already received similar but externally performed therapy were excluded.

The retrospective data analysis was approved by the Ethics Committee of the Faculty of Medicine of the Sigmund Freud University (Vienna, Austria; ECNr: 804-2023) and was part of the thesis by co-author (E.H.). The legal guardian(s) signed consent forms for each surgical procedure. Informed consent for this study was waived due to the retrospective nature of the study. The study adhered to the principles of the Declaration of Helsinki, local legislation and institutional requirements, and followed the Strengthening the Reporting of Observational Studies in Epidemiology (STROBE) guidelines (26).

### 2.2 Data collection

Demographic data including age (in years), gender (female or male), height (in cm), weight (in kg), and reported allergies/intolerances were extracted. The body mass index (BMI) was calculated as weight in kg divided by the square of height in m (kg/m^2^). Percentile curves from the Centers for Disease Control and Prevention (CDC) (2000) for girls and boys were used to obtain BMI percentiles. The BMI-for-age charts considering the child's age, height, weight, and gender were used to obtain the BMI percentiles for each patient. The following categories were used to group BMI percentiles: underweight (< 5th percentile), healthy weight (5th−84th percentile), overweight (85th−94th percentile), obesity (≥95th percentile), and severe obesity (120% of ≥95th percentile greater or BMI ≥35 kg/m^2^).

Intervention related data included details regarding adverse events (AE). The procedure and some data from three cases were previously reported (27, 28). All procedures were only performed on children who had been referred to us with an external diagnosis of ASD and were in accordance with Austrian regulations and relevant medical guidelines and regulations. All SCTs were performed as single procedures in a class IIa operating room with sterile air flow according to the following standard operating procedure (SOP): (1) anesthesia was prepared with rectal administration of Midazolam (1 mg/kg body weight with max. of 15 mg); (2) slowly starting sedation with 5–8 ml (i.v.) 1%-Propofol (sedoanalgesia); (3) positioning of the patient on one side following surgical washing and draping the anterior and posterior iliac crest; (4) injection of 2 ml of 1%-Xylocaine at the planned puncture sites on the periosteum and subcutaneously; (5) aspiration of BM from the posterior and anterior iliac crest followed by a transfer of the BM aspirate to a sterile blood bag; (6) the BM aspirate was then processed in the operating room according to the proprietary protocol using a fully automated cell separator (Sepax S-100; Biosafe S.A., Eysins, CH); (7) after lumbar puncture of the dural sac 2 ml of cerebral spinal fluid (CSF) was routinely withdrawn in order to prevent high intrathecal pressure secondary to injection of the stem cell concentrate; (8) intrathecal administration of the obtained BM concentrate (~1 ml/10 kg body weight); and (9) i.v. administration of the remaining BM concentrate and plasma supernatant (27, 28). Standard postoperative care was applied.

Details regarding intra- (on the day of intervention) and post-intervention (day 1 to 7 days post-intervention and/or to last follow-up) adverse events (AEs)–including type of AE, duration (from onset to resolution), seriousness (not serious/serious AE), intensity (mild/moderate/severe/life-threatening/death-related AE), relation to treatment (not related/unlikely/possible/probable/definite/unknown), and action taken (no action taken/treatment adjusted/temporarily interrupted/treatment permanently discontinued due to this AE/concomitant medication taken/non-drug therapy given/hospitalization/prolonged hospitalization)—were extracted from patient's file from a standardized AE form.

Efficacy was evaluated according to the parents‘/guardians'-generated Autism Treatment Evaluation Checklist (ATEC) score. The 77-item ATEC was developed by the Autism Research Institute (San Diego, CA) (6) to describe changes over time. Total ATEC scores range from 0 to 179 points and are determined by the sum of four sections, including subgroup I: speech/language/communication (14 items; 0–2 points each, total 0–28 points); subgroup II: sociability (20 items; 0–2 points each, total 0–40 points); subgroup III: sensory/cognitive awareness (18 items; 0–2 points each, total 0–36 points); and subgroup IV: health/physical/behavior (25 items; 0–3 points each, total 0–75 points). A higher ATEC score indicates a higher severity of ASD symptoms/impairment, and a lower score indicates lower severity of ASD symptoms/impairment. Total ATEC scores were grouped according to Mahapatra et al. (29) into severe (80–179 points), moderate (50–79 points), mild (21–49 points), and a group with no or very minimal signs of autism (0–20 points).

### 2.3 Statistical analysis

Patients' characteristics were presented using descriptive statistics. Qualitative data were expressed by numbers and percentages, and quantitative data as means with standard deviation (SD) or median and range (minimum to maximum). For the primary outcome (ATEC score), only complete data sets were included in the analysis to ensure consistency and avoid bias. For secondary outcome variables, missing data were addressed in the results section.

Data distribution was inspected by visual inspection of the histograms and the Kolmogorov Smirnov tests. The Wilcoxon signed rank tests were used for comparisons of ATEC scores before and after an intervention. Comparisons of the ATEC scores between two independent groups (girls/boys) were performed using Mann-Whitney U tests. Kruskal-Wallis tests and Bonferroni *post hoc* pairwise tests were used to examine ATEC score differences between the three independent age groups. ATEC scores obtained at three different time points were compared by Friedman tests and *post hoc* analysis were conducted using Wilcoxon signed-rank tests and with a Bonferroni correction applied. Two independent categorical parameters were assessed using Chi-square tests. Effect size was calculate according to Cohen's d for paired samples (*d*_diff_ = mean_diff/_SD_diff_). Values of *d*_diff_ around 0.2 were considered small, around 0.5 medium, and 0.8 or higher large in magnitude.

Statistical significance was considered when the *p*-value was < 0.05 (two-sided). The Statistical Package for the Social Sciences (SPSS 26.0 Package Facility; SPSS Inc., Chicago, IL, United States) software was used for data analysis.

## 3 Results

### 3.1 Patient characteristics at baseline

A total of 176 patient charts were reviewed for eligibility. Of these, 44 (Supplementary Table S1) were excluded due to missing or incomplete primary endpoint data (ATEC score) and four patients who have already received similar but externally performed therapy were also not included. The remaining 128 charts met inclusion criteria. Patient characteristics at baseline of all included patients (*n* = 128) and evaluated charts are presented in Table 1. There were almost four times more males (79%) than females (21%) in the cohort. Comparisons of characteristics between girls and boys were similar, except for height, with boys being significantly taller than girls (Supplementary Table S2).

**Table 1:** Patient characteristics at baseline.

**Characteristics^1^**	**ALL (*n* = 128)**
Age (years) mean ± SD [median (min–max)]	6.4 ± 3.3 [6 (2–16)]
Age groups (n,%) pre-school age (2–5 years) school-age (6–12 years) teenagers (13–18 years)	63 (49.2%)58 (45.3 %)7 (5.5%)
Gender (*n*, %) female male	27 (21.1%)101 (78.9%)
Height^2^ (cm) mean ± SD [median (min-max)]	121.8 ± 20.0 [117.5 (83–180)]
Weight (kg) mean ± SD [median (min-max)]	27.6 ± 16.4 [22 (10–98)]
BMI^1^ mean ± SD [median (min-max)]	17.5 ± 4.3 [16.2 (11.0–31.5)]
BMI percentiles^1^ mean ± SD [median (min-max)]	55.5 ± 36.0 [62.5 (1–100)]
BMI percentile groups^1^ (n,%) Underweight (< 5th percentile) Healthy Weight (5th−84th percentile) Overweight (85th−94th percentile) Obesity (≥95th percentile) Severe Obesity (120% of ≥95th percentile greater or BMI ≥35 kg/m^2^)	15 (12.1%) 67 (54.0%) 17 (13.7%) 17 (13.7%) 8 (6.5 %)
Allergies/Intolerances^3^ Diet-related (*n* = 25) Dairy products/casein, lactose (*n* = 9) Cereals/gluten (*n* = 7) Histamine (*n* = 1) Specific foods [soy (*n* = 1), honey (*n* = 1), nuts (*n* = 1), fish (*n* = 1), mushrooms (*n* = 1), tomatoes (*n* = 1); others unspecified (*n* = 2)] Medication (*n* = 14) Antibiotics (*n* = 11) Specific drugs [benzodiazepine (*n* = 1), corticosteroid (*n* = 1), NSAID (*n* = 1)] Other (*n* = 9) allergic reactions to Pollen (*n* = 2) Insect stings (*n* = 2) allergens causing dermatitis (*n* = 2) Dust (*n* = 1) Mold (*n* = 1) Plasters (*n* = 1)	

BMI, body mass index; NSAID, nonsteroidal anti-inflammatory drug; SD, standard deviation.

1 At time of 1st procedure.

2 Height was not available in four cases.

3 Information regarding allergies/intolerances was not reported or available in 87 cases (68.0%). Six of the 42 patients reported two different allergies/intolerances.

### 3.2 Safety

A total of 32.8% of the 128 children who underwent intrathecal injection of the bone marrow concentrate two times also had a third (*n* = 34, 26.6%), fourth (*n* = 7, 5.5%), or fifth (*n* = 1, 0.8%) treatment. Hence, the total number of interventions was 307.

Not a single intra- nor post-interventional serious AE was reported in 307 procedures performed for 128 children with ASD. More than half of all patients (51.6%; *n* = 66) showed not even one AE after any of their procedures. All reported AEs were recorded as being “possible” or “probable” in relation to the intervention and resolved spontaneously either with no action taken, with medication given, or with non-drug therapy given. Table 2 shows all AEs which occurred after the first (in 28.9% of patients), second (in 21.9% of patients) or third (in 11.9% of patients) treatments. No AEs were reported after the fourth and fifth treatments. Nausea and/or vomiting, which was most likely due to intrathecal application, was the most often reported AE. Almost all AEs (97.8%) were either mild (*n* = 45) or moderate (*n* = 43) events.

**Table 2:** Details of all minor and transient adverse events detected after bone marrow aspiration with intrathecal injection of bone marrow concentration in children with autism spectrum disorder.

**Type of AE**			**Number of AEs after the 1st procedure in 37 of 128 patients**	**Number of AEs after the 2nd procedure in 28 of 128 patients**	**Number of AEs after the 3rd procedure in 5 of 42 patients**
**Cell- related AEs**		Hyperactivity/agitation	3	1	0
**Procedure-related AEs**	**Anesthesia**	Fatigue	2	0	0
		Vertigo	3	0	0
		Dehydration	1	1	0
	**Puncture site** [BMA + LP]	Pain	8	3	1
		Hematoma/bleeding	3	0	0
	**Intrathecal-application**	Headache	10	5	3
		Vomiting/nausea	17	21	2
		Fever	2	1	0
**Other AEs**		Gastric pain (due to pain killer)	1	0	0
		Numbness of left foot	1	0	0
		Local swelling at puncture site	0	1	0
**TOTAL**			**51**	**33**	**6**
AE intensity (mild/moderate/severe)			29 / 22 / 0	16 / 15 / 2	0 / 6 / 0

AE, adverse event; BMA, bone marrow aspiration; LP, lumbar puncture.

### 3.3 Efficacy

The time between the first (before) and second (after) intervention was 12.7 ± 7.0 months. The TOTAL and all subgroup ATEC scores as well as the severity groups significantly decreased showing the degree of improvement after the first intervention (Table 3).

**Table 3:** Autism Treatment Evaluation Checklist (ATEC) changes.

**ATEC**	**Before intervention (prior 1st intervention) (*n* = 128)**	**After intervention (prior 2nd intervention) (*n* = 128)**	***p*-value^1^**
Speech/language/communication (0–28 points)	18.0 ± 6.2 20 (1–28)	14.6 ± 6.7 16 (0–25)	< 0.001
Sociability (0–40 points)	15.8 ± 7.8 16 (0–34)	10.6 ± 5.7 10 (0–25)	< 0.001
Sensory/cognitive awareness (0–36 points)	17.3 ± 7.0 18 (1–34)	11.9 ± 6.0 12 (0–31)	< 0.001
Health/physical/behavior (0–75)	21.1 ± 11.5 19.5 (2–57)	16.1 ± 10.0 14 (0–48)	< 0.001
TOTAL score (0–179 points)	72.2 ± 24.6 72 (26–129)	53.3 ± 21.3 52 (10–98)	< 0.001
Severity groups 0 (no: < 20 points) 1 (mild: 20–49 points) 2 (moderate: 50–79 points) 3 (severe: 80–179 points)	2.2 ± 0.7 2 (1–3)	1.6 ± 0.7 2 (0–3)	< 0.001

ATEC, Autism Treatment Evaluation Checklist.

^1^Wilcoxon signed-rank test.

**Figure 1:**
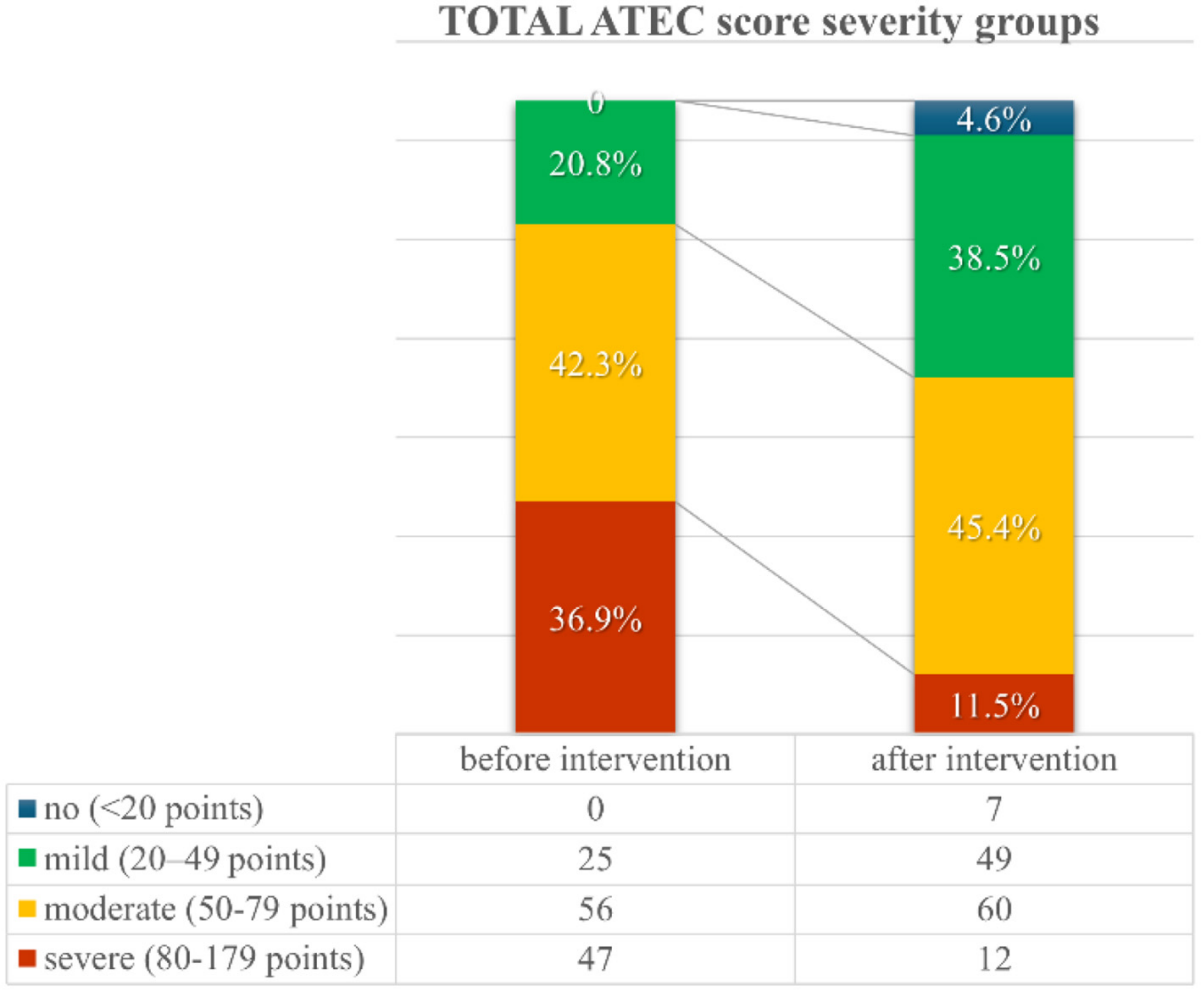
TOTAL Autism Treatment Evaluation Checklist (ATEC) score severity groups distribution from before to after the first intervention.

Following the intervention, almost 5% of patients initially categorized as “mild” (*n* = 4) or “moderate” (*n* = 3) shifted to the ‘no symptoms' category of the total ATEC score severity group (Figure 1). This and a 25.4% reduction/reassignment from the initially categorized severe group (*n* = 47) to a milder symptom category suggests a measurable improvement in autism-related symptoms. Further details regarding distribution shifts from before to after the intervention regarding total ATEC score severity groups are presented in Supplementary Table S3.

**Figure 2:**
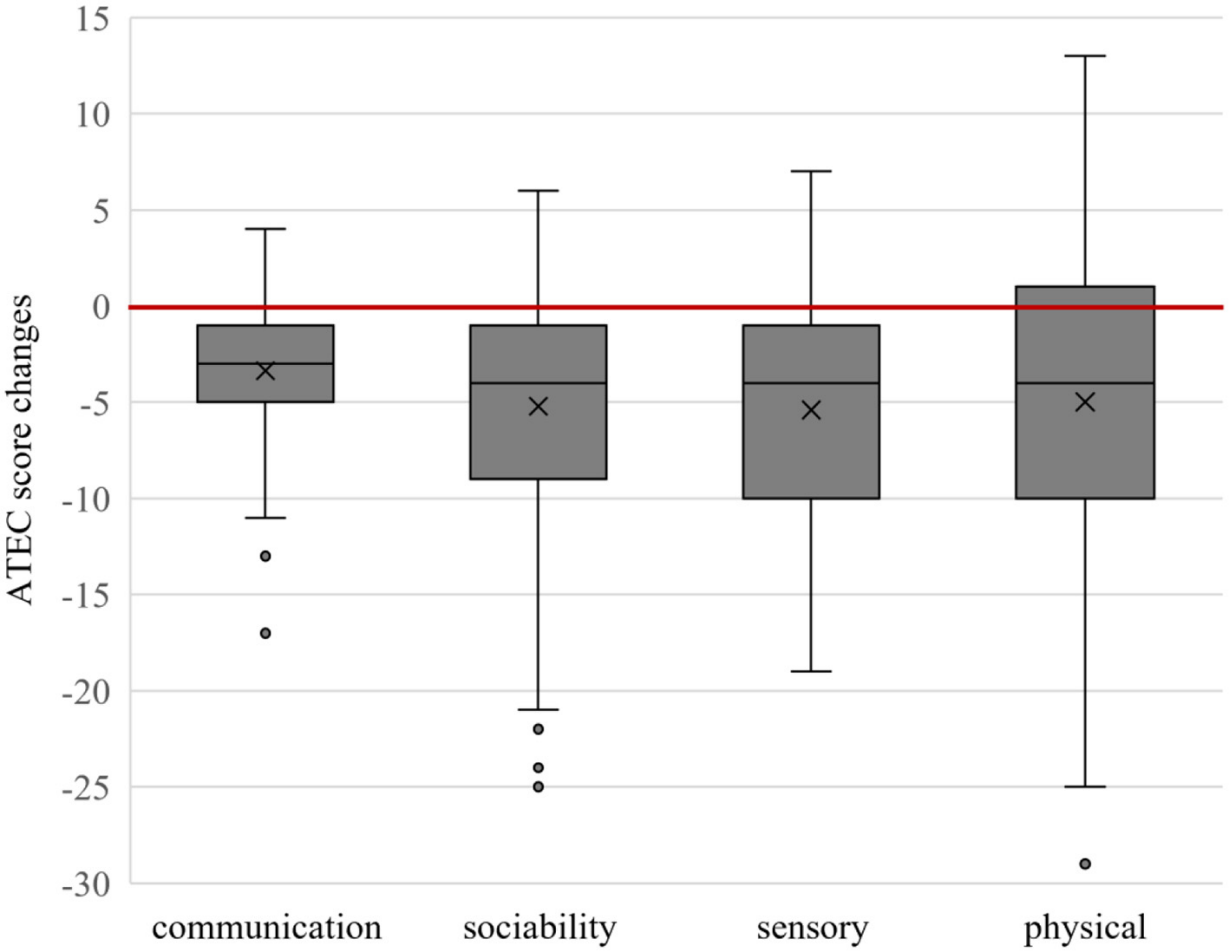
Box plot of Autism Treatment Evaluation Checklist (ATEC) subgroup score changes. Below the red line shows improvement.

The mean changes in the overall ATEC score were −19.0 ± 17.1 (median −16 range, −60 to 9; with a very large effect size, *d*_diff_ = 1.11) and the subgroups I (speech/language/communication:−3.4 ± 3.5; median −3 range, −17 to 4; with a large effect size, *d*_diff_ = 0.97), II (sociability: −5.2 ± 6.1; median−4 range, −25 to 6; with a large effect size, *d*_diff_ = 0.85), III (sensory/cognitive awareness:−5.4 ± 5.7; median −4 range, −19 to 7; with a large effect size, *d*_diff_ = 0.95), and IV (health/physical/behavior: −5.0 ± 8.7; median −4 range, −29 to 13; with a medium effect size, *d*_diff_ = 0.57) are shown in Figure 2.

**Figure 3:**
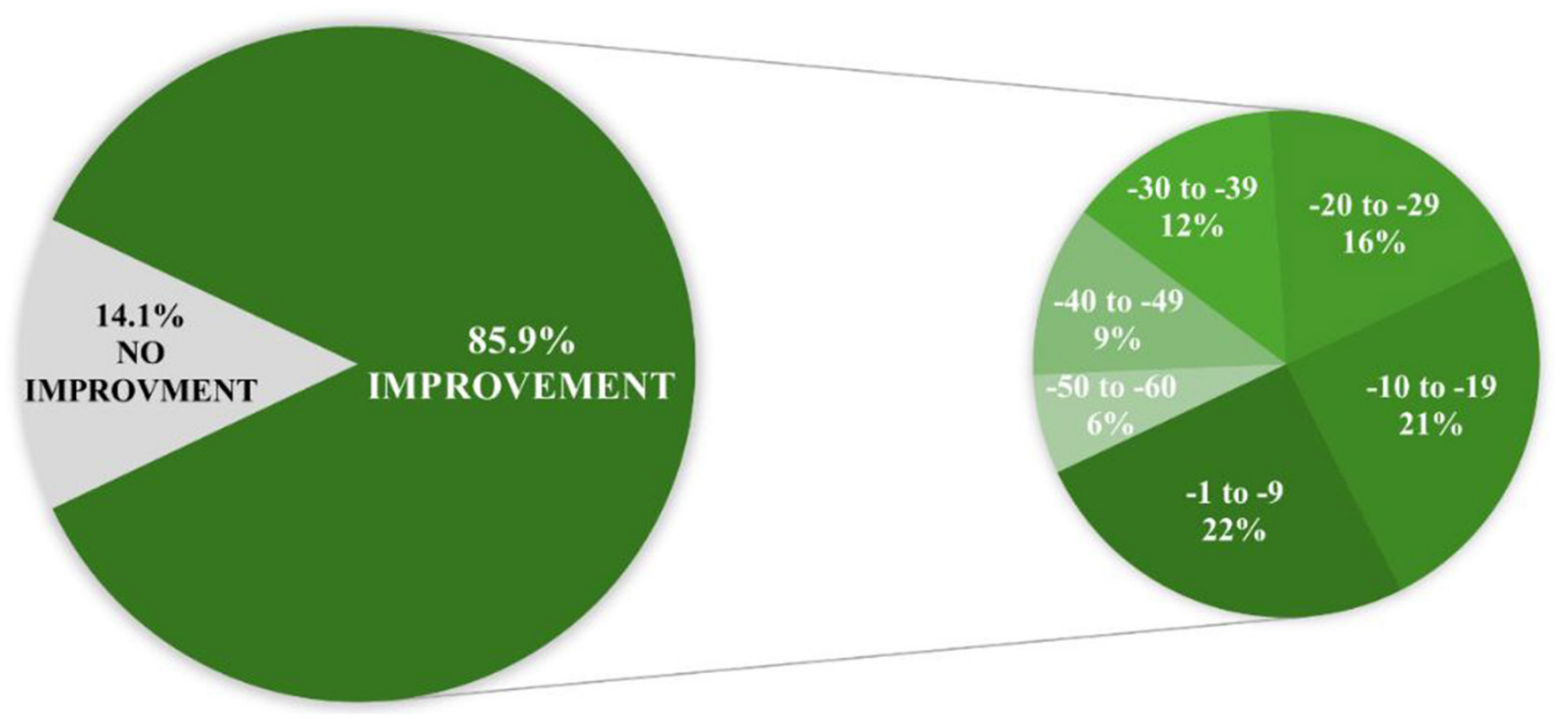
Pie chart of Autism Treatment Evaluation Checklist (ATEC) point improvements.

In the majority of patients, the ATEC TOTAL score decreased from 1 to even 60 points from before to after the first intervention. In only 18 of the 128 cases the ATEC TOTAL score did not improve and showed no or minor changes from 0 to +9 points from the 1st to the 2nd procedure (Figure 3).

### 3.4 Comparison between girls and boys

Both genders showed significant improvements from before to after the intervention with one exception in the girls' group (ATEC subgroup III: physical). Significantly higher post-interventional ATEC scores were detected regarding the subgroup II (sociability) and IV (physical) in the girls group. However, ATEC changes and ATEC severity groups were also similar between genders. All ATEC comparisons are presented in Supplementary Table S4.

### 3.5 Comparison between age groups

Significant ATEC-improvements from before to after the intervention were detected in preschoolers and school-aged children, but not in all subgroups of the teenagers (Supplementary Table S5). However, comparisons regarding ATEC improvements and ATEC severity groups were not significantly different between the age groups. *Post-hoc* analyses revealed a statistically significant difference between preschoolers and school-aged children regarding the initial ATEC subgroup I (communication), indicating that younger children have lower communication skills; *post-hoc* analysis also revealed ATEC score differences in subgroup III, indicating less sensory improvements in children between 6 and 12 years compared to preschoolers.

### 3.6 Subgroup analysis of patients with three interventions

**Figure 4:**
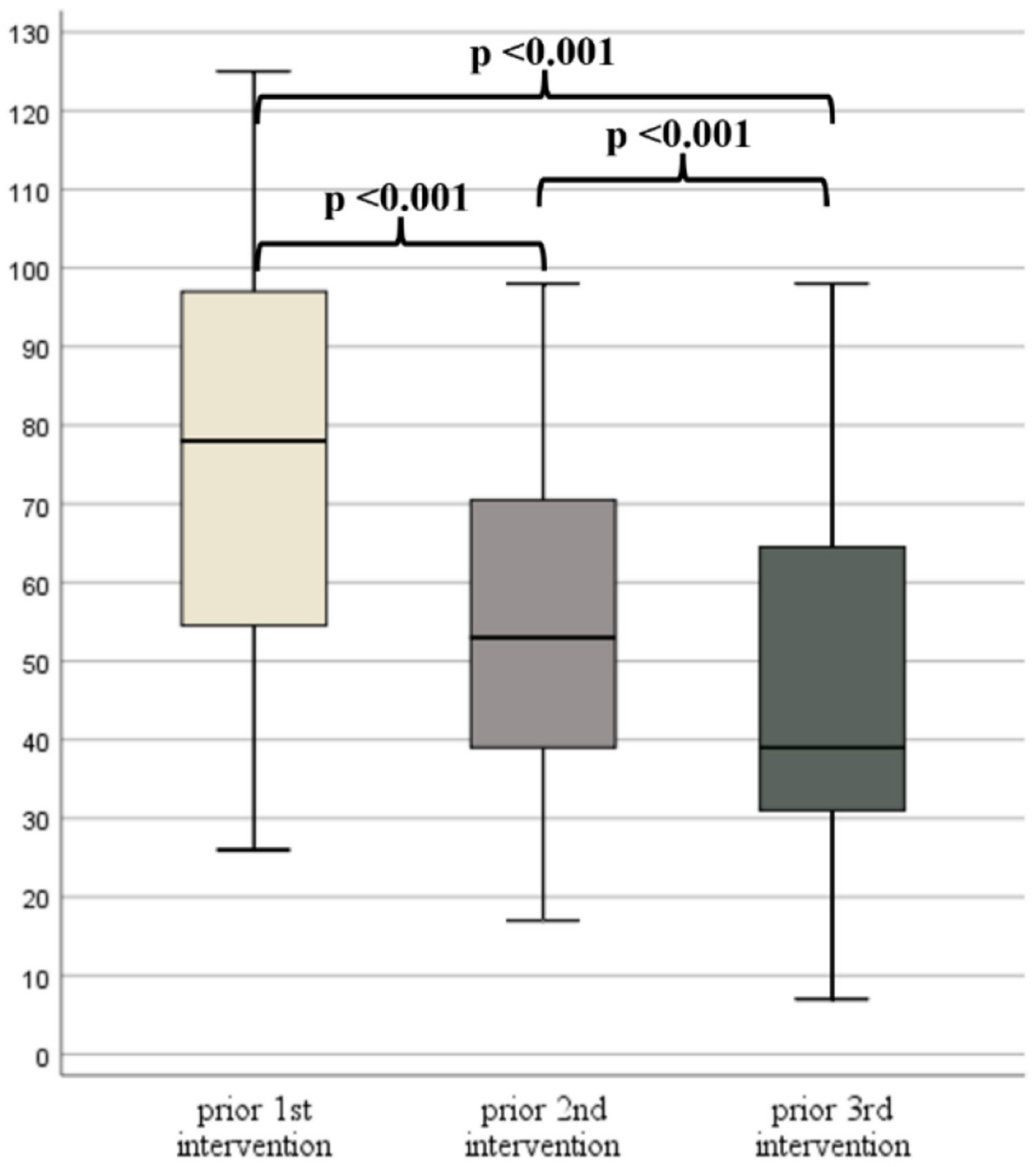
Box plots of Autism Treatment Evaluation Checklist (ATEC) TOTAL scores prior to the 1st, 2nd, and 3rd intervention.

The time between the first, second and third interventions in 39 patients who received three interventions, and who had ATEC scores available (92.9% of the 42 patients), was 10.0 ± 2.3 months and 23.0 ± 5.5 months, respectively. There was a statistically significant difference in all ATEC score subgroups and in the total ATEC scores (Figure 4) between the three time points (Supplementary Table S6). However, according to *post-hoc* analyses, no significant differences were detected between the second and third intervention regarding subgroup I and III (Supplementary Table S6).

### 3.7 Subgroup analysis of patients with more than three interventions

**Figure 5:**
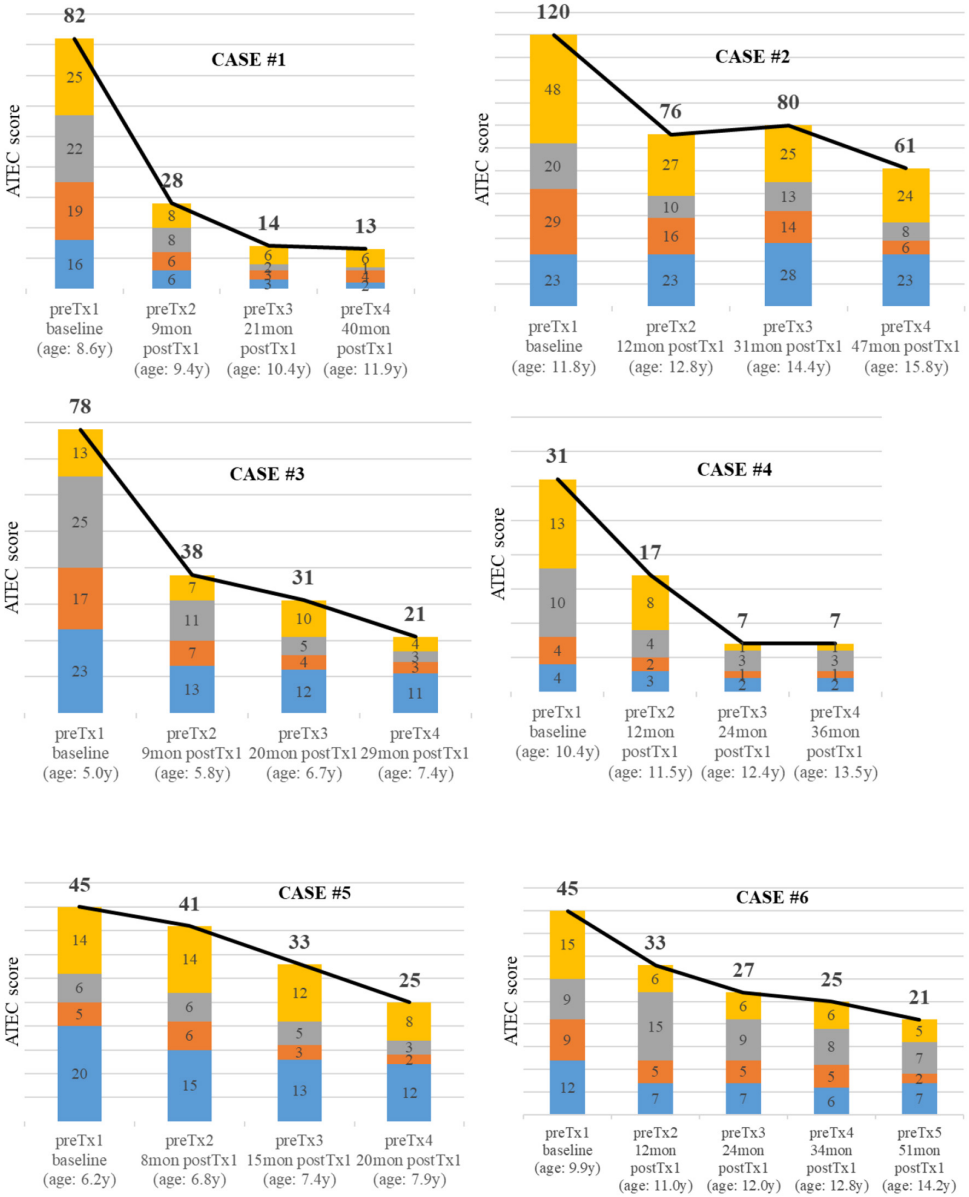
Autism Treatment Evaluation Checklist (ATEC) scores prior each intervention of six cases with more than three interventions. The blue category indicates the subgroup speech/communication, orange the subgroup sociability, gray the subgroup sensory/cognitive awareness, and yellow the subgroup health/physical/behavior.

All ATEC scores prior to each intervention of the five patients (case #1 to #5) who underwent four and the one patient with five treatments (case #6) are shown in Figure 5. One of the patients with a fourth intervention had no ATEC score prior to the last intervention. The highest improvements occurred after the first intervention, continued to improve over time, and remained reduced even three to four years after the first intervention.

## 4 Discussion

Overall, according to our findings, autologous bone marrow aspiration with intrathecal injection of bone marrow concentrate interventions yielded significant symptom improvements in children with ASD. Total ATEC scores showed an average reduction of 19 points, with some cases achieving a 60-point decrease within 1 year. Approximately 5% of the children with ASD showed no or very minimal signs of autism after the first procedure. Improvements were found regardless of age and gender. Not a single serious adverse event was observed and the other AEs (e.g., nausea/vomiting) were resolved within a week after the procedure.

Our findings concerning safety (i.e., no serious adverse event) and efficacy (i.e., clinical improvement) are in accordance with other clinical studies which investigated cell-based therapies, as reported in systematic reviews with meta-analyses (30, 31). In particular, clinical studies by Sharma et al. (25, 32), Sharifzadeh et al. (22) and Nguyen et al. (23, 33) investigated autologous bone marrow derived and intrathecally applied procedures. While Sharma et al. (32) (*n* = 254; 34.0 to 29.7; *p* < 0.001) and Nguyen et al. (23) (*n* = 30; 50 range, 40–55.5 to 47.3 range, 35.5–53.5; *p* < 0.050) found significant improvements regarding Childhood Autism Rating Scale (CARS) scores from baseline to follow-up (7.5 and 12 moths, respectively), Sharifzadeh et al. (22) (*n* = 32; 36.4 ± 3.6 to 30.8 ± 5.0; *p* = 0.102) did not in the 12-month follow-up. It seems that the use of cultured cells in the protocol by Sharifzadeh et al. (22), in contrast to the uncultured cell preparations employed by Sharma et al. (32) and Nguyen et al. (23), may have contributed to the divergent outcomes observed across the studies. The most recent phase-II clinical trial by Nguyen et al. (33) showed significant differences in post-interventional CARS scores between the intervention (40.6 ± 4.9) and the control group (43.7 ± 5.5; *p* = 0.046). Although the CARS (a diagnostic tool) is not directly comparable to the ATEC (a tool to measure progress), it can be assumed that our results are similar to the results of the above-mentioned publications–especially the study with a large sample size (32)–due to the significant correlation between CARS and ATEC (34, 35).

Furthermore, the clinical study by Sharma et al. (32) demonstrated that improvements were independent of age and gender, a finding that aligns with our results. The female-to-male ratio of this study was higher (ratio 7.2:1) than ours (ratio 3.7:1). Historically, the male-to-female ratio has been estimated at 4:1, but newer numbers suggest it may be closer to 3:1, according to the Centers for Disease Control and Prevention (CDC) (36). However, the prevalence between genders was not the subject of both investigations.

Sharma et al. (22) was able to achieve slightly higher overall improvements regarding the Indian Scale for Assessment of Autism (ISAA; 94.5%) and CARS (95.3%) when compared to our study which uses the ATEC score (85.9%). The reason for this approximately 10% difference might be variations regarding the intervention protocol (e.g., administration of granulocyte colony stimulating factor (GCSF) injections prior to bone marrow aspiration) or score sensitivities. However, no study has achieved a 100% improvement with autologous bone marrow derived and intrathecally applied procedures. We assume that each intervention targets specific symptoms of this heterogeneous disease. If no improvement is observed, additional interventions may be required to address the remaining aspects of ASD.

Cell therapies have shown potential in alleviating symptoms of ASD, yet the precise mechanisms underlying these promising outcomes continue to be widely debated. It is hypothesized that autologous bone marrow-derived stem cell transplantation may engage specific mechanisms when administered intrathecally. The following section outlines several hypothetical mechanisms through which stem cell interventions may exert their therapeutic effects. Although not yet empirically confirmed, these proposed pathways draw upon current biological insights and are intended to serve as a conceptual basis for future experimental validation. It is important to note that these mechanistic considerations were not part of the present study and are provided solely for theoretical context. The bone marrow aspirate is a heterogenous mix of cells including hematopoietic stem cells (HSC) and mesenchymal stem cells (MSC). Bone marrow-derived cell concentrates also contain other key components, such as growth factors, cytokines, stem cell derived exosomes (37), and other extracellular vehicles (EVs). The following mechanisms may be involved: (a) immune modulation: transplanted cells may regulate the immune response by reducing excessive inflammation and excitotoxic damage caused by overactive immune cells (38, 39); (b) modulation of inflammation: transplanted cells, especially when applied intrathecally, influence microglia and astrocytes, mitigating their pro-inflammatory and excitotoxic effects. Research suggests that immune reconstitution following autologous hematopoietic stem cell transplantation plays a role in restoring immune balance, which could be relevant in conditions involving immunoexcitotoxicity (40–43); (c) neuroprotection: transplanted cells release growth factors and anti-inflammatory cytokines, and thus may protect neurons from further damage; (d) neuroregeneration: stem cells may promote the repair and regeneration of damaged neural tissues by differentiating into neuronal or glial cells. They may also enhance neuronal communication by supporting synaptic remodeling and axonal regeneration (44). Furthermore, MSCs naturally secrete brain-derived neurotrophic factor (BDNF), which contributes to promoting neuron survival and plasticity. Indeed, a preclinical study suggested that BDNF-mediated neurogenesis may be a key mechanism behind the observed behavioral changes, supporting the idea that BDNF-linked stem cell therapy could be a promising method to treat ASD (45); and (d) angiogenesis: some studies suggest that stem cells can promote the formation of new blood vessels, improving oxygen and nutrient supply to affected areas (46).

## 5 Limitations

One might argue that the ATEC score decreases over time without specific interventions. Indeed, longitudinal changes of ATEC score as a function of age was reported by a previous publication (29). They showed that the total ATEC score decreased by approximately 28 (2 to 3-year-old group), 20 (3 to 6-year-old group), and 14 (6 to 12-year-old group) points over a 2-year period, which was similar to our findings of 21 (2 to 5-year-old group) and 16 (6 to 12-year-old group) points. However, the time interval between the first and second intervention was in most of our cases (93.0%) around one year (median 11 range, 4–22). The mean total ATEC difference of those patients with a time interval of 12 months or less also showed high improvements in both age groups (2 to 5-year-old group: −21.9 ± 17.0 and 6 to 12-year-old group: −16.2 ± 18.4), indicating that the reduction in ATEC score is more likely due to the intervention rather than age. Another point of discussion regarding the ATEC as an outcome tool might be the fact that the ATEC is a parent-rated measure. However, previous studies found strong correlations between the ATEC and the professionally administered CARS (34, 35). However, parent-rated assessments such as the ATEC, and even professionally administered tools like the CARS, provide valuable insights despite being prone to subjectivity. Indeed, the recent phase II trial by Nguyen et al. (33) provides compelling evidence for the therapeutic potential of intrathecal bone marrow cell administration in children with ASD, demonstrating sustained improvements across multiple validated scales including the CARS at 12 months, and thereby reinforcing the plausibility of stem cell efficacy beyond confounding factors such as natural disease progression or scale variability. It remains an imperative to identify and develop objective methods of measurement—such as biomarkers—so as to accurately diagnose and monitor progress in children with ASD. However, recent evidence from Maric et al. (47) suggests that specific cerebrospinal fluid cytokine profiles—particularly elevated baseline IL-27 levels—may serve as predictive biomarkers for clinical response to intrathecal bone marrow aspirate concentrate therapy in children with ASD, thereby offering preliminary support for objective biological measures in monitoring therapeutic outcomes.

Several other limitations warrant attention. Concomitant therapies may have been subject to under- or overreporting due to reliance on caregiver-reported outcomes, potentially affecting the interpretation of treatment effects. Moreover, the small sample sizes within specific subgroups—such as adolescents and female participants—limit the generalizability of the findings. The variable duration between interventions. Comorbidities as possible confounding factors have not been investigated in depth. Our long-term findings, extending up to 3–4 years, appear promising; however, they are based on a very limited number of patients, which is a limiting factor. Therefore, long-term outcomes should be systematically investigated in future studies. Finally, causal relationships cannot be definitively established, as the observed outcomes may have been influenced by placebo effects or natural developmental progression.

Future research is essential for developing and validating reliable, standardized objective approaches which minimize bias and enhance precision in evaluation. To address the limitations of our current findings, future research should incorporate several methodological enhancements. First and foremost, conducting a randomized controlled clinical trial is essential to rigorously evaluate the efficacy of the presented intervention. The integration of objective biomarkers and/or functional neuroimaging modalities would provide mechanistic insights and substantiate observed functional changes. Employing psychometric assessments administered independently by external professionals may increase the objectivity and reliability of outcome measures. Finally, analyses should also account for potential confounding variables, including intervention duration, baseline severity, and comorbid conditions, to enhance the interpretability of treatment effects.

The retrospective design of this study, along with the absence of a control group, limits the ability to draw definitive conclusions regarding long-term safety and effectiveness. However, its large sample size enhances the reliability of the findings, contributes to and confirms previous clinical studies, and provides valuable insights for future research.

Altogether, stem cell-based procedures seem to be superior to psychological treatment alone. However, according to Nguyen et al. (33) the combination of both approaches may be the best choice for children with ASD and their families.

## 6 Conclusion

In summary, our findings show that autologous bone marrow aspiration with intrathecal injection of bone marrow concentrate interventions in children with ASD are safe and effective in reducing the ATEC-score significantly–and thus, improving symptoms of ASD.

## Data Availability

The original contributions presented in the study are included in the article/Supplementary material, further inquiries can be directed to the corresponding author.
