# Supplementary material for: Intrathecal injection of bone marrow concentrate in children with autism spectrum disorder: a retrospective chart analysis
Source: Front Med (Lausanne). 2025 Sep 18;12:1666486. doi: 10.3389/fmed.2025.1666486 (PMC12488664; doi:10.3389/fmed.2025.1666486)
Supplement: Supplementary file 1 [file Data_Sheet_1.pdf]

Frontiers in Medicine ORIGINAL RESEARCH article

Supplementary Material

Intrathecal injection of bone marrow concentrate in children with autism spectrum disorder: A retrospective chart analysis

Georg S. Kobinia[1,2*], Adam Bukaty[2], Elisabeth Holly[3], Gloria Kobinia[1,2], Philipp R. Heuberer[1,4], Brenda Laky[1,2,3,4]

[1]Austrian Society of Regenerative Medicine, Vienna, Austria

[2]Kobinia-Med, Institute for Regenerative Medicine, Vienna, Austria

[3]Sigmund Freud University, Faculty of Medicine, Vienna, Austria

[4]Austrian Research Group for Regenerative and Orthopedic Medicine (AURROM), Vienna, Austria

*** Correspondence:**
Univ.Doz. Georg S. Kobinia, MD
georg.kobinia@regmedaustria.org

Georg S. Kobinia: georg.kobinia@regmedaustria.org ORCID: 0000-0002-3814-4718

Adam Bukaty: adamjoel@rocketmail.com

Elisabeth Holly: elisabeth.holly@aon.at

Gloria Kobinia: publicaffairs@regmedaustria.org

Philipp R. Heuberer: philipp@heuberer.at ORCID: 0000-0001-6684-1978

Brenda Laky: brenda.laky@regmedaustria.org ORCID: 0000-0003-1198-4132

Supplement Table S1. Comparison of patients not in study due to incomplete Autism Treatment Evaluation Checklists (ATEC) (n=44) to patients in the study (n=128).

Characteristics[a]	in study (n=128)	not in study (n=44)	p-value
Age (years) mean ± SD [median (min-max)]	6.4 ± 3.3 [6 (2-16)]	7.1 ± 3.7 [6 (2-17)]	0.359 [a]
Gender (n, %) female / male	27 (21.1%) / 101 (78.9%)	2 (4.5%) / 42 (95.5%)	0.010 [b]
**ATEC before treatment**	**n=128**	**n=14**	
Speech/language/communication (0-28 points)	18.0 ± 6.2 [20 (1-28)]	17.3 ± 6.2 [17.5 (8-27)]	0.617 [a]
Sociability (0-40 points)	15.8 ± 7.8 [16 (0-34)]	17.9 ± 8.7 [16.5 (8-37)]	0.476 [a]
Sensory/cognitive awareness (0-36 points)	17.3 ± 7.0 [18 (1-34)]	20.1 ± 5.7 [19 (12-31)]	0.164 [a]
Health/physical/behavior (0-75 points)	21.1 ± 11.5 [19.5 (2-57)]	25.8 ± 12.3 [27.5 (2-44)]	0.108 [a]
TOTAL score (0-179 points)	72.2 ± 24.6 [72 (26-129)]	81.0 ± 27.2 [77 (36-136)]	0.253 [a]
**ATEC after treatment**	**n=128**	**n=20**	
Speech/language/communication (0-28 points)	14.6 ± 6.7 [16 (0-25)]	11.3 ± 7.9 [9 (2-25)]	0.080 [a]
Sociability (0-40 points)	10.6 ± 5.7 [10 (0-25)]	9.2 ± 6.8 [8 (0-31)]	0.143 [a]
Sensory/cognitive awareness (0-36 points)	11.9 ± 6.0 [12 (0-31)]	11.5 ± 7.8 [10 (1-31)]	0.398 [a]
Health/physical/behavior (0-75 points)	16.1 ± 10.0 [14 (0-48)]	14.7 ± 8.6 [13 (0-27)]	0.690 [a]
TOTAL score (0-179 points)	53.3 ± 21.3 [52 (10-98)]	46.6 ± 26.2 [35.5 (10-107)]	0.104 [a]

[a]Mann-Whitney U test. [b]Fisher's exact test.

This is a provisional file, not the final typeset article

Supplement Table S2. Patients' characteristics – comparisons between girls and boys

Characteristics*	girls (n=27)	boys (n=101)	p-value
Age (years)			
mean ± SD	5.7 ± 3.6	6.6 ± 3.1	0.065[d]
median (min-max)	5 (2-16)	6 (2-16)	
Age groups			
pre-school age (2-5 years)	14 (51.9%)	49 (48.5%)	0.802[e]
school-age (6-12 years)	11 (40.7%)	47 (46.5%)	
teenagers (13-18 years)	2 (7.4%)	5 (5.0%)	
Height[a] (cm)			
mean ± SD	113.5 ± 17.9	124.2 ± 20.0	0.011[d]
median (min-max)	115 (83-170)	120 (86-180)	
Weight (kg)			
mean ± SD	22.8 ± 10.1	28.9 ± 17.5	0.073[d]
median (min-max)	21 (13-62)	22 (10-98)	
BMI[a]			
mean ± SD	17.0 ± 2.4	17.6 ± 4.7	0.578[d]
median (min-max)	16.4 (12.9-22.9)	16.1 (11.0-31.5)	
BMI percentiles[a]			
mean ± SD	62.0 ± 29.0	53.7 ± 37.6	0.484[d]
median (min-max)	63 (1-99.8)	62 (1-100)	
BMI percentile groups[a]			
Underweight (<5th percentile)	1 (3.7%)	14 (14.4%)	0.286[e]
Healthy Weight (5th - 84th percentile)	18 (66.7%)	49 (50.5%)	
Overweight (85th - 94th percentile)	5 (18.5%)	12 (12.4%)	
Obesity (≥95th percentile)	2 (7.4%)	15 (15.5%)	
Severe Obesity (120% of ≥95th percentile greater or BMI ≥35 kg/m^2)	1 (3.7%)	7 (7.2%)	
Allergies/Intolerances[c]			
Diet-related (n=25)	**6 (75.0%)**	**19 (47.5%)**	
Dairy products / casein, lactose	2	7	
Cereals / gluten	2	5	
Histamine	0	1	
Specific foods	2	6	
[soy (f: n=1), honey (m: n=1), nuts (m: n=1), fish/cod (f: n=1), mushrooms (m: n=1), tomatoes (m: n=1); others unspecified (m: n=2)]			
Medication (n=14)	**1 (12.5%)**	**13 (32.5%)**	
Antibiotics	1	10	0.353[e]
Specific drugs	0	3	
[benzodiazepine (n=1), corticosteroid (n=1), NSAID (n=1)]			
Other (n=9) allergic reactions to	**1 (12.5%)**	**8 (20.0%)**	
Pollen	1	1	
Insect stings	0	2	
allergens causing dermatitis	0	2	
Dust	0	1	
Mold	0	1	
Plasters	0	1	

Abbreviations: BMI, body mass index; f, female; m, male; NSAID, nonsteroidal anti-inflammatory drug; SD, standard deviation.

[a]At time of 1st procedure.

[b]Height was not available in four male cases.

[c]Information regarding allergies/intolerances was not reported or available in 20 girls (15.6%) and 67 males (52.3%). Six males and one female of the 42 patients reported two different allergies/intolerances.

[d]Mann-Whitney test. [e]Chi-Square test.

Supplement Table S3. Shift of Autism Treatment Evaluation Checklist (ATEC) severity groups.

TOTAL ATEC score groups			After intervention (prior 2nd intervention)				TOTAL
			no	mild	moderate	severe	
**Before intervention** (prior 1st intervention)	female	mild	1	3	0	0	**4**
		moderate	1	3	8	0	**12**
		severe	0	2	3	6	**11**
		TOTAL female	2	8	11	6	**27**
	male	mild	3	16	2	0	**21**
		moderate	2	21	21	0	**44**
		severe	0	4	26	6	**36**
		TOTAL male	5	41	49	6	**101**
	all	mild	4	19	2	0	**25**
		moderate	3	24	29	0	**56**
		severe	0	6	29	12	**47**
**TOTAL**			**7**	**49**	**60**	**12**	**128**

Abbreviations: ATEC, Autism Treatment Evaluation Checklist.

Supplement Table S4. Comparisons between girls and boys regarding Autism Treatment Evaluation Checklist (ATEC)

ATEC	girls (n=27)	boys (n=101)	p-value (between group)
Communication (0-28 points)			
before	19.9 ± 5.4 [20.5 (9 – 28)]	17.5 ± 6.3 [20 (1 - 26)]	0.106[b]
after	15.2 ± 6.1 [17 (4 - 25)]	14.5 ± 6.9 [16 (0 - 25)]	0.765[b]
p-value (within group)	<0.001[a]	<0.001[a]	
changes	-4.7 ± 4.0 [-4 (-17 – 0)]	-3.0 ± 3.3 [-3.0 (-13 – 4)]	0.063[b]
Sociability (0-40 points)			
before	17.9 ± 7.8 [17 (4 - 33)]	15.3 ± 7.8 [15 (0 - 34)]	0.156[b]
after	12.9 ± 6.9 [15 (0 - 23)]	10.0 ± 5.2 [9 (1 - 25)]	0.020[b]
p-value (within group)	<0.001[a]	<0.001[a]	
changes	-5.0 ± 6.3 [-3 (-21 – 6)]	-5.3 ± 6.1 [-4.0 (-25 – 6)]	0.646[b]
Sensory (0-36 points)			
before	18.1 ± 7.0 [18 (7 - 30)]	17.1 ± 7.1 [17 (1 - 34)]	0.545[b]
after	12.5 ± 6.9 [12 (1 - 31)]	11.8 ± 5.7 [11 (0 - 27)]	0.702[b]
p-value (within group)	<0.001[a]	<0.001[a]	
changes	-5.7 ± 6.4 [-4 (-19 – 5)]	-5.3 ± 5.5 [-4.0 (-19 – 7)]	0.833[b]
Physical (0-75)			
before	23.6 ± 13.6 [22 (3 - 49)]	20.4 ± 10.8 [19 (2 - 57)]	0.367[b]
after	19.8 ± 11.0 [20 (0 - 48)]	15.1 ± 9.5 [13 (0 - 40)]	0.035[b]
p-value (within group)	0.100[a]	<0.001[a]	
changes	-3.8 ± 9.8 [-2 (-22 – 13)]	-5.3 ± 8.4 [-5.0 (-29 – 13)]	0.356[b]
TOTAL score (0-179 points)			
before	79.5 ± 26.2 [77 (35 - 129)]	70.3 ± 23.9 [69 (26 - 126)]	0.128[b]
after	60.4 ± 25.8 [69 (10 - 98)]	51.3 ± 19.6 [51 (13 - 98)]	0.060[b]
p-value (within group)	<0.001[a]	<0.001[a]	
changes	-19.1 ± 19.6 [-9 (-56 – 7)]	-18.9 ± 16.4 [-17.0 (-60 – 9)]	0.706[b]
Severity groups (0=no/1=mild/ 2=moderate/3=severe)			
before	0 / 4 (14.8) / 12 (44.4) / 11 (40.7)	0 / 21 (20.8) / 44 (43.6) / 36 (35.6)	
	2.3 ± 0.7 [2 (1 – 3)]	2.1 ± 0.7 [2.0 (1 – 3)]	0.498[b]
after	2 (7.4) / 8 (29.6) / 11 (40.7) / 6 (22.2)	5 (5.0) / 41 (40.6) / 49 (48.5) / 6 (5.9)	
	1.8 ± 0.9 [2 (0-3)]	1.6 ± 0.7 [2 (0-3)]	0.183[b]
	0.004[a]	<0.001[a]	

Abbreviations: ATEC, Autism Treatment Evaluation Checklist.

[a]Wilcoxon Signed Ranks Test
[b]Mann-Whitney U Test

Supplement Table S5. Comparisons between age groups regarding Autism Treatment Evaluation Checklist (ATEC)

ATEC	**Pre-school age (2-5 years)** (n=63)	**School-age (6-12 years)** (n=58)	**Teenagers (13-18 years)** (n=7)	**p-value** (between group)
Communication (0-28 points)				
before	19.8 ± 5.3 [21 (4 - 28)][i]	16.2 ± 6.3 [17 (1 - 27)][i]	16.7 ± 9.1 [20 (6 - 27)]	0.006[b]
after	15.7 ± 6.3 [17 (0 - 25)]	13.6 ± 6.7 [14.5 (1 - 25)]	12.7 ± 9.5 [15 (1 - 23)]	0.232[b]
p-value (within group)	<0.001[a]	<0.001[a]	0.018[a]	
changes	-4.0 ± 3.9 [-4 (-17 - 3)]	-2.6 ± 3.1 [-2 (-13 - 4)]	-4.0 ± 2.2 [-4 (-7 - -1)]	0.059[b]
Sociability (0-40 points)				
before	16.7 ± 7.4 [17 (0 - 34)]	14.7 ± 8.1 [15 (1 - 34)]	17.6 ± 9.1 [15 (6 - 33)]	0.228[b]
after	10.8 ± 6.1 [10 (0 - 25)]	10.4 ± 5.3 [10 (1 - 22)]	10.6 ± 6.6 [8 (4 - 23)]	0.936[b]
p-value (within group)	<0.001[a]	<0.001[a]	0.018[a]	
changes	-5.9 ± 6.2 [-5 (-22 - 5)]	-4.3 ± 6.3 [-3 (-25 - 6)]	-7.0 ± 3.3 [-7 (-11 - -2)]	0.103[b]
Sensory (0-36 points)				
before	18.2 ± 7.1 [19 (1 - 32)]	16.4 ± 6.9 [15.5 (6 - 34)]	17.4 ± 6.8 [16 (7 - 27)]	0.235[b]
after	11.7 ± 6.6 [11 (0 - 27)]	12.3 ± 5.0 [12 (1 - 31)]	10.7 ± 8.0 [11 (0 - 21)]	0.710[b]
p-value (within group)	<0.001[a]	<0.001[a]	0.051[a]	
changes	-6.5 ± 6.0 [-5 (-19 - 7)][ii]	-4.1 ± 5.0 [-3 (-15 - 3)][ii]	-6.7 ± 6.4 [-6 (-13 - 5)]	0.036[b]
Physical (0-75)				
before	20.6 ± 11.4 [18 (4 - 49)]	21.6 ± 11.9 [20 (2 - 57)]	20.6 ± 9.5 [23 (6 - 35)]	0.910[b]
after	16.2 ± 10.9 [14 (0 - 48)]	16.1 ± 9.0 [14.5 (3 - 40)]	14.6 ± 9.8 [12 (3 - 28)]	0.893[b]
p-value (within group)	0.001[a]	<0.001[a]	0.063[a]	
changes	-4.4 ± 8.6 [-4 (-29 - 13)]	-5.5 ± 9.1 [-4.5 (-24 - 13)]	-6.0 ± 6.6 [-7 (-15 - 5)]	0.708[b]
TOTAL score (0-179 points)				
before	75.3 ± 23.2 [75 (30 - 129)]	68.8 ± 25.9 [66.5 (26 - 128)]	72.3 ± 25.4 [77 (39 - 103)]	0.270[b]
after	54.5 ± 23.0 [53 (10 - 98)]	52.4 ± 18.8 [52 (13 - 96)]	48.6 ± 26.5 [52 (22 - 91)]	0.782[b]
p-value (within group)	<0.001[a]	<0.001[a]	0.018[a]	
changes	-20.8 ± 16.6 [-18 (-59 - 6)]	-16.4 ± 18.1 [-11.5 (-60 - 9)]	-23.7 ± 9.8 [-23 (-34 - -7)]	0.122[b]
Severity groups (0=no/1=mild/2=moderate/3=severe)				
before	0 / 9 (14.3) / 27 (42.9) / 27 (42.9)	0 / 14 (24.1) / 27 (46.6) / 17 (29.3)	0 / 2 (28.6) / 2 (28.6) / 3 (42.9)	
after	2.3 ± 0.7 [2 (1 - 3)]	2.1 ± 0.7 [2 (1 - 3)]	2.1 ± 0.9 [2 (1 - 3)]	0.215[b]
	4 (6.3) / 23 (36.5) / 27 (42.9) / 9 (14.3)	3 (5.2) / 23 (39.7) / 30 (51.7) / 2 (3.4)	0 / 3 (42.9) / 3 (42.9) / 1 (14.3)	
	1.7 ± 0.8 [2 (0 - 3)]	1.5 ± 0.7 [2 (0 - 3)]	1.7 ± 0.8 [2 (1 - 3)]	0.720[b]
	<0.001[a]	<0.001[a]	0.083[a]	

Abbreviations: ATEC, Autism Treatment Evaluation Checklist.

[a]Wilcoxon Signed Ranks Test

[b]Kruskal-Wallis test. Post-hoc tests using Dunn's test with Bonferroni correction showed the significant differences between preschoolers and schoolers regarding (i) the initial ATEC subgroup I communication (adjusted p = 0.004) and (ii) ATEC score differences in subgroup III (adjusted p = 0.047).

Supplement Table S6. Autism Treatment Evaluation Checklist (ATEC) of 39 patients with three interventions

ATEC	prior 1st intervention	prior 2nd intervention	prior 3rd intervention	p-value[a]	p-value[b]
Speech/language/communication (0-28 points)	17.7 ± 6.4<br>20 (4-27)	14.3 ± 6.5<br>15 (1-25)	13.4 ± 6.4<br>13 (2-28)	<0.001	<0.001[i]<br><0.001[ii]<br>0.058[iii]
Sociability (0-40 points)	16.7 ± 8.7<br>16 (4-33)	11.4 ± 6.3<br>11 (2-25)	8.4 ± 6.3<br>7 (0-23)	<0.001	<0.001[i]<br><0.001[ii]<br><0.001[iii]
Sensory/cognitive awareness (0-36 points)	17.7 ± 6.1<br>18 (6-30)	11.6 ± 5.4<br>11 (0-24)	10.3 ± 5.9<br>9 (0-25)	<0.001	<0.001[i]<br><0.001[ii]<br>0.051[iii]
Health/physical/behavior (0-75)	22.8 ± 12.2<br>23 (2-57)	17.1 ± 10.0<br>16 (3-48)	14.3 ± 10.7<br>12 (0-44)	<0.001	<0.001[i]<br><0.001[ii]<br>0.009[iii]
TOTAL score (0-179 points)	74.8 ± 26.2<br>78 (26-125)	54.3 ± 21.6<br>53 (17-98)	46.4 ± 23.4<br>39 (7-98)	<0.001	<0.001[i]<br><0.001[ii]<br><0.001[iii]

Abbreviations: ATEC, Autism Treatment Evaluation Checklist.

[a] Friedman test.

[b]Post hoc analysis with Wilcoxon signed-rank tests were conducted with a Bonferroni correction applied, resulting in a significance level set at $p < 0.017$. P-values from post-hoc analyses between the (i) 1st and 2nd, (ii) 1st and 3rd, and (iii) 2nd and 3rd treatment.
